# Analyzing and Predicting User Participations in Online Health Communities: A Social Support Perspective

**DOI:** 10.2196/jmir.6834

**Published:** 2017-04-24

**Authors:** Xi Wang, Kang Zhao, Nick Street

**Affiliations:** ^1^ Interdisciplinary Graduate Program in Informatics The University of Iowa Iowa City, IA United States; ^2^ Department of Management Sciences The University of Iowa Iowa City, IA United States

**Keywords:** social support, machine learning, community networks, patient engagement, prediction

## Abstract

**Background:**

Online health communities (OHCs) have become a major source of social support for people with health problems. Members of OHCs interact online with similar peers to seek, receive, and provide different types of social support, such as informational support, emotional support, and companionship. As active participations in an OHC are beneficial to both the OHC and its users, it is important to understand factors related to users’ participations and predict user churn for user retention efforts.

**Objective:**

This study aimed to analyze OHC users’ Web-based interactions, reveal which types of social support activities are related to users’ participation, and predict whether and when a user will churn from the OHC.

**Methods:**

We collected a large-scale dataset from a popular OHC for cancer survivors. We used text mining techniques to decide what kinds of social support each post contained. We illustrated how we built text classifiers for 5 different social support categories: seeking informational support (SIS), providing informational support (PIS), seeking emotional support (SES), providing emotional support (PES), and companionship (COM). We conducted survival analysis to identify types of social support related to users’ continued participation. Using supervised machine learning methods, we developed a predictive model for user churn.

**Results:**

Users’ behaviors to PIS, SES, and COM had hazard ratios significantly lower than 1 (0.948, 0.972, and 0.919, respectively) and were indicative of continued participations in the OHC. The churn prediction model based on social support activities offers accurate predictions on whether and when a user will leave the OHC.

**Conclusions:**

Detecting different types of social support activities via text mining contributes to better understanding and prediction of users’ participations in an OHC. The outcome of this study can help the management and design of a sustainable OHC via more proactive and effective user retention strategies.

## Introduction

### Overview

Nowadays more and more people use the Internet to satisfy their health-related needs. According to a study by the Pew Research Center, 80% of adult Internet users in the United States use the Internet for health-related purposes. Among them, 34% read health-related experiences or comments from others [1]. Online health communities (OHCs) offer a venue for people to interact with peers facing similar health problems. Modern OHCs have incorporated many ways for communication and health management, such as listserv, discussion forums, private messaging, chat rooms, blogs, friend subscriptions, health tracking tools, and so on. OHCs range from general-purpose communities, such as MedHelp and PatientsLikeMe, to those dedicated to a specific health issue, such as diabetes and smoking. Many OHCs host their own websites, whereas some are built on existing social networking services, such as Facebook. Many studies have revealed the advantages and disadvantages of OHCs compared with traditional offline support groups [2]. Although OHCs may face problems such as sporadic membership of active users, inaccurate information, deception, and insincerity of strangers [3-5], they also offer tremendous benefits such as broad reach, availability, and anonymity. Consequently, OHCs have gained popularity in recent years, and it is estimated that 5% of all Internet users participated in an OHC [6].

Studies of user behaviors in OHCs are valuable in several ways. First, outcomes of these studies can inform better management and design of a successful OHC, which can help to promote new treatments and healthy lifestyles and reveal adverse drug effects [7-9]. Like other online communities, successful OHCs would like to encourage users’ participations and prevent users’ churn (ie, leaving a community), because one of the keys for the success of an online community is active participations from and relationship building among its members [10,11]. In contrast, poor participations and transient membership can lead to the termination or failure of an online community [12]. Specifically, OHCs will not be sustainable if its users keep leaving because most of the social support can only be provided by active members of the OHC.

Second, a successful and sustainable OHC will provide more benefits to individual users. This is because a user’s continued participation in an OHC can be helpful and therapeutic [13-18]. On one hand, receiving such support can be empowering [19] and help patients adjust to the stress of living with and fighting against their diseases [20,21]. The support they receive online can also improve their offline life and health management [22]. On the other hand, besides receiving support from others, staying in an OHC and providing support to others can be beneficial to providers as well [20]. There is actually a positive relationship between posting frequency and psychosocial well-being [23]. In other words, a user’s continued participation in an OHC can help herself or himself as well as others. Admittedly, for some individuals who have received satisfactory support from an OHC or recovered from the disease, leaving the OHC may not be a bad thing for themselves. However, even though user-generated information about a disease will still be available on the Internet to new OHC members, most of the psychosocial benefits for individual users cannot be achieved if the exodus of experienced users in the OHC keeps happening, leaving new members stranded [23]. In fact, providing assistance for new members from experienced members and reminding members to participate continuously are also key factors for the success of online communities [12]. Therefore, better understanding and accurate prediction of users’ participations in OHCs can help to build and sustain a successful OHC through improved community design, management, and user retention.

As social support is a pillar of OHCs, a natural question to ask would be: when it comes to users’ participations, are a user’s Web-based activities in different types of social support related to her or his continued participation in an OHC? If so, can we predict whether and when a user will churn from an OHC based on these social support activities? Despite the large amount of research on social support in OHCs, few studies have answered this question systematically by examining users’ seeking, receiving, and provision of various types of social support from large-scale datasets. An explanatory model [24] suggested that receiving more emotional support is associated with users’ longer stay in an OHC. However, the types of social support investigated were limited and only the receiving of support was considered, while we mentioned earlier that providing social support is also important and beneficial. Analyzing large-scale data from a real-world OHC with various data analytics techniques, including text mining, survival analysis, and predictive modeling, our research explained as well as predicted users’ continued participations in OHCs from the perspective of online social support.

### Background and Research Goals

#### Social Support and OHCs

According to Shumaker and Brownell [25], social support refers to the “exchange of resources between at least two individuals perceived by the provider or the recipient to be intended to enhance the well-being of the recipient.” Based on the nature of exchanged “resources,” community psychology researchers have identified different types of social support [26,27]. In this research, we adopted the four types of social support proposed in [28,29]: informational support, emotional support, companionship, and instrumental support. Informational support is the transmission of information, suggestion, or guidance to the community users [30]. The content of such a post in an OHC is usually related to advice, referrals, education, and personal experience with the disease or health problem. Example topics include side effects of a drug, ways to deal with a symptom, experience with a physician, or medical insurance problems. Emotional support, as its name suggests, contains the expression of understanding, encouragement, empathy, affection, affirming, validation, sympathy, caring, and concern. Companionship, also known as network support, consists of chatting, humor, teasing, as well as discussions of offline activities and daily life that are not necessarily related to one’s health problems. Thus, they are sometimes referred to as “off-topic” discussions. Examples include sharing jokes, birthday wishes, holiday plans, or Web-based scrabble games. Instrumental support, or tangible support, refers to offline support activities in the physical world, such as transporting others to hospitals, assistance in grocery shopping, and so forth. Empirical studies suggested that informational support, emotional support, and companionship are common in many OHCs, but instrumental support is rare, as such support is limited by geographical proximity [31,32]. Also, the further exchange and arrangement of instrumental support may often occur via private or offline communication channels (eg, setting a time for grocery shopping via cell phones). To simplify our automated social support classification, we did not consider instrumental support in this study.

The emergence of OHCs provides new opportunities to study social support at unprecedented scales and granularities. Traditional studies on offline support communities studies relied heavily on data collected through ethnographical observations, interviews, questionnaires, or surveys [14,33-36]. However, research using these data collection methods faces 3 challenges. First, the scale of the data is limited because observations and interviews are labor intensive and time consuming. Second, results may be biased due to the realities of sampling community members. For example, members who are active in or satisfied with their communities may be more likely to respond to questionnaires or surveys. Third, survey and interview methods typically have coarse temporal granularity and rely on members’ recall of past events and associated feelings. This makes it very difficult to accurately track community members’ activities during an extended time period.

By contrast, OHCs not only enable but also record asynchronous and distributed social interactions among individuals, making the “big data” available for computational analysis. Such detailed data of users’ online interactions (eg, the amount, content, and time of interactions) contain valuable information on users’ behaviors. To study social support at such a large scale and fine granularity, we need to reveal the nature of social support embedded in users’ contributions in an automated way. Hence, our first research goal was about mining large-scale text data contributed by OHC users to detect different types of social support activities.

Goal #1: Detect the seeking and provision of different types of social support from unstructured text of large-scale distributed interactions among OHC users.

#### Online Community Participations

According to Preece [37], an online community is a group of people who are connected through the Internet and interact over time around a shared purpose, interest, or need. The success of online communities depends largely on sustained participations and voluntary contributions from users [38]. Researchers have revealed factors related to user participation in online communities, such as open-source software development [39], Wikipedia [40], and Question & Answering communities [41,42].

Different from other types of online communities, seeking and obtaining various types of social support is a key reason people participate in an OHC [43]. On one hand, OHC users have a common identity as the patient of a disease, and information about the disease will be discussed and exchanged very often. On the other hand, the exchange of emotional support and participations in companionship, often in the form of seemingly off-topic discussions, can help OHC users get to know each other personally as they share things beyond health and the common disease. To understand which types of social support are more indicative of user engagement in the community, our second research goal was to run an explanatory model to connect different types of social support with user continued participation.

Goal #2: Develop an explanatory model to explore whether users’ activities in seeking, providing, and receiving different types of social support are related to their continued participations in an OHC.

#### Churn Predictions

In addition to building an explanatory model to understand factors related to users’ continued participations, another key to sustain an online community is to predict user churn, so that the community can intervene when a user is about to churn and try to retain her or him. Implications for churn prediction are not limited to online communities, but also to other online and offline businesses, such as telecommunication [44], retail [45], Internet access service [46], and online gaming [47]. These models have leveraged different types of data about customers and the market, including those related to money, contracts, demographics, usage, products, complaints, competitions, and social networks [48-50].

When it comes to online communities, traditional churn prediction faces challenges as well as opportunities. On one hand, many of the features commonly used for churn prediction in for-profit business are not available or make no sense. For instance, users’ demographic data (eg, residential address, income, and ethnicity) is usually unavailable or inaccurate in online communities. Also, because many online communities are based on voluntary participations and do not charge any fee, monetary and contractual issues become largely irrelevant. On the other hand, online communities provide more detailed data about users’ behaviors for predictive analytics [51]. While previous churn prediction studies have leveraged structured data of users’ activities, few have examined the unstructured content of users’ interactions or contributions. In contrast, in many online communities, including OHCs, large amount of such content is publicly available from the Web. Previous research on online social networks and social media has suggested that content analysis can be helpful in areas such as personalized recommendation [52], community discovery [53], and influential user identification [54]. We believe analyzing unstructured text posted by online community users from a social support perspective should contribute to accurate churn prediction in OHCs.

Moreover, many churn predictions for traditional business are limited to snapshot data—a model is learned from data for customers, who were active during a specific period (ie, the training period, usually a couple of months to half a year), based on which customers churned in the subsequent testing period (often a few months). For an online community, data for a user’s complete “life span” in the community can be available for analysis. Such complete data can provide valuable information because those who churn after the first week may behave differently from those who churn after a month. Thus, our last research goal is about building a predictive model using data of users’ social support activities.

Goal #3: Leverage data about users’ Web-based social support activities over time to build a predictive model to forecast whether and when a user will churn from an OHC.

## Methods

In this research, we used the data from a very popular peer-to-peer OHC (Breastcancer.org) among breast cancer survivors as a case study. We designed a Web crawler to collect data from its online forum. Our dataset consisted of all the public posts and basic user profile information from October 2002 to August 2013.

### Methods of Social Support Detection

As we mentioned earlier, informational support, emotional support, and companionship are the three major types of social supports in OHCs. Thus for each post from an OHC, we need to determine whether it was seeking informational support (SIS), providing informational support (PIS), seeking emotional support (SES), providing emotional support (PES), or simply about companionship (COM). Note that we did not differentiate the seeking and provision of companionship because the nature of companionship was about participation and sharing. By getting involved in activities or discussions about companionship through posting, one was seeking and providing support at the same time. It was also possible that a post could belong to more than 1 of the aforementioned categories. Table 1 lists example posts for each category and a post that belongs to 2 categories.

**Table 1:** Example posts for different types of social support.

Social support category	Examples
Companionship (COM)	(1) *Kelly Have a wonderful time in Florida, enjoy the sun and fun. Heather* (2) *I’m loving her new CD. Didn’t recognize any of the songs at first, but there are a few now that I find myself singing the rest of the day.* (3) *This game has the poster making a new 2 word phrase starting with the second word of the last post Example: Post : Hand out Next poster: Outcast Next poster: Cast Iron Next poster: Iron Age Now let’s begin the game~ Age Old*
Seeking informational support (SIS)	*Where do you buy digestive enzymes and what are they called?*
Seeking emotional support (SES)	*I feel like everyone else's lives are going forward, they have plans, hopes, aspirations because they feel. I am one of those not yet out of the woods. I was also someone who could never get cancer. I was a good person, exercised, ate well. Good people don't get sick. I have taken the step of antidepressants, they mitigate the damage, but do not block the pain or sadness I feel.*
Providing informational support (PIS)	*I had surgery Aug05 for bc recurrance. B4 surgery I had 33 IMRT rads, prior to that had 4A/C &amp; 4 Taxol. I had bc in 2000 &amp; had 37 rads in same general area. Now, my surgery won't heal. Wound doc says there is adema or something on my sternum (shown on recent MRI). My wound has been draining since it broke open in Sept.*
Providing emotional support (PES)	*Hope you feel better soon, we are here! Prayers Hugs come from Massachusetts APPLE♥* *.*
Providing informational support (PIS) and providing emotional support (PES)	*I am also the daughter of a 35 yrs BC survivor. Mom is just now going through some more Cancer - alas - they found it in her lung, but it is totally unlikely to be a follow-up of her old BC. I am 45, and was 43 at DX time, my mom was diagnosed at 38... and I am a BRCA2 carrier. Tina, one day at a time. Maybe you'll get good news - it is so hard to wait!!! It is also important to remember that - whatever it is, it is highly treatable, and that YOU WILL SURVIVE too!!! and life goes on after. It will take some time, but it goes on... see my picture? even the hair is back!!! Hugs to all. I am happy you all found your way here, it is a great site for exchanging information, learning and finding support.*

Because it is practically impossible to label all 2.8 million posts manually, we used text classification algorithms to decide what kinds of social support each post contained. Text mining techniques have been adopted to analyze large-scale text data from online social networks, including texts from online health communities (similar findings by Ko D-G, Mai F, and Zhe S, unpublished data, 2015). To train a text classification algorithm, we leveraged human annotated data. We randomly selected 1333 posts out of our dataset. After being trained on the definitions and examples of the aforementioned 5 categories of social supports (SIS, PIS, SES, PES, and COM), 5 human annotators were asked to read each post and decide whether the post belongs to one or more categories of social support (See Multimedia Appendix 1 for the training instruction). To control the quality of human annotations, we also added to the pool 10 posts that had been annotated by domain experts. For each post, we only accepted results from annotators whose performance on the 10 quality-control posts was among the top 3. The results from the other 2 annotators were discarded. Then, a majority vote among the top 3 annotators was used to determine whether a post was related to a category of social support. Multimedia Appendix 1 lists the outcome of the annotation.

Users in OHCs may have different writing styles or linguistic preferences to express themselves. To capture these characteristics, we examined each post and extracted various types of features for training the classifier: basic features, lexical features, sentiment features, and topic features. Multimedia Appendix 2 includes more details about the feature engineering for social support classification.

### Methods of Participation Analysis

After detecting the nature of social support in each post, we conducted survival analysis to study how different types of social support activities were related to users’ participations. An individual may enter or exit a community not only based on his or her own expectations and behaviors, but also based on the community’s reactions toward this individual [55]. Thus, in addition to users’ own posting behaviors, we also examined whether the receiving or exposure to different types of social support would impact a user’s participation.

Our survival analysis was based on the Cox proportional-hazards model [56], which assessed the importance of different independent variables on the “survival time” it takes for a specific event to occur (Multimedia Appendix 3 includes more details of the model setup). Specifically, for our analysis, an “event” referred to a user’s cessation of activities in the OHC (ie, churn from the OHC). A user’s survival time was measured from the difference between her last and first posts in the OHC. Similar to a previous study [24], we assumed that a user had churned from this OHC if she had no post during the last 12 weeks in our dataset. For those who were active in the OHC during the last 12 weeks, their survival time was right-censored because they were still participating in this OHC.

Table 2 summarizes independent variables in our model. They reflect users’ own posting behaviors in various social support categories, as well as the amount of social support they received in threaded discussions in direct or indirect ways. A user received support directly when she initiated a thread to seek support and got support from others’ replies to the thread. Meanwhile, social support could also be received indirectly when one replied to a thread started by another user because she might be exposed to support that other users provided to the original poster. In addition to these independent variables, we also included 3 control variables to reflect users’ overall levels of activities.

The experiment included 19,165 users whose time spans of activities in the OHC exceeded 1 month. Values of control and independent variables were collected based on their behaviors in seeking, providing, and receiving social support in the first month of their participations. To reduce the impact of multi-collinearity, we calculated the correlation coefficients for every pair of variables. We then removed TotalPost and NumThread from the model, as both were strongly correlated with the other control variable InitPost (with correlation coefficients greater than .8). Thus, our model for survival analysis included 1 control variable and 10 independent variables.

### Methods of Churn Prediction

If different types of social support activities are indeed related to users’ participations in OHCs, OHC managers can design more effective interventions to retain users. Such interventions can be more targeted when OHC mangers know who are likely to leave and when. Therefore, this section proposes a model to predict whether and when a user will churn from an OHC and demonstrates the value of including social support activities over time in such predictions.

**Table 2:** Control variables and independent variables in the survival analysis.

Variables	Descriptions
TotalPost^a^	The total number of posts a user has published (excluded from the model due to strong correlation with *InitPost*)
InitPost^a^	The total number of threads a user initiated
NumThread^a^	The number of threads a user contributed to (excluded from the model due to strong correlation with *InitPost*)
PES^b^	The number of a user’s posts that provided emotional support
PIS^c^	The number of a user’s posts that provided informational support
SES^d^	The number of a user’s posts that sought emotional support
SIS^e^	The number of a user’s posts that sought informational support
COM^f^	The number of a user’s posts that were related to companionship
RIS_D_	Direct informational support received—the number of informational support posts a user received after initiating a support-seeking thread.
RES_D_	Direct emotional support received—the number of emotional support posts a user received after initiating a support-seeking thread.
RIS_I_^g^	Indirect informational support received—the number of informational support posts a user was exposed to in threads that she or he did not initiate but contributed to.
RES_I_^g^	Indirect emotional support received—the number of emotional support posts a user was exposed to in threads that she or he did not initiate but contributed to.
RCOM^g^	Companionship received—the number of companionship posts a user was exposed to in threads that she or he did not initiate but contributed to.

^a^denotes the three control variables.

^b^PES: providing emotional support.

^c^PIS: providing informational support.

^d^SES: seeking emotional support.

^e^SIS: seeking informational support.

^f^COM: companionship.

^g^For *RIS*_I_, *RES*_I_, and *RCOM*, we assumed that a user read others’ replies that were posted within 7 days before the user’s replies in the same thread.

Basic features for our predictive model are derived from the 13 independent variables we used for survival analysis (Table 2). Because these features aggregated users’ activities during the training period, we also measured how users’ values on the 13 features varied over time using four types of temporal features. Specifically, for each user, we divided her activities measured by each of the 13 basic features into weeks and used 4 additional metrics to capture how the value of each feature changes over the weeks, including overall slope, Shannon entropy, stability, and temporal variations (TV) as proposed in [57]. In addition to cumulative values for each basic feature during the training period, we also conjectured that a user’s intention to churn might be better captured during the last week of her online activities. Thus, we also included values for basic features during the last week of the training period if the training period was longer than 1 week. Each basic feature for the last week also had 4 corresponding features to reflect its temporal patterns (ie, slope, Shannon entropy, stability, and TV), although the unit of time was day instead of week. We also added into the feature set the time difference between a user’s registration time and the time of her first post because it might reveal what brought the user to the OHC for the first time. A user who is eager to find some information might have a low gap between the registration time and the time of first posting. More details of features are presented in Multimedia Appendix 4.

In terms of modeling the churn prediction problem, a user was said to churn in her *k-th* week if her last online activity occurred during her *k-th* week in the OHC. Similar to our hazard model, users whose last online activities occurred during the last 12 weeks in our dataset were not considered as churned. To predict whether a user would churn in the *k-th* week of her online activities, we focused on all users who were still active before the *k-th* week and extracted data based on their *k* weeks of activities. For example, the dataset for predicting user churn during the third week contains users who were still active in the OHC before their third week of online activities. Data of their behaviors during their first 2 weeks were collected for training. Users who churned in their third week and never came back were labeled as “positive” instances in the dataset.

Previous studies indicate that different predictive models for each time period may not be an efficient solution. If the OHC wanted to know who would churn in their second and third weeks, 2 models were needed. Inspired by [58], we tried to consolidate all predictive models for churn in different weeks into 1 unified model by leveraging a user’s social support activities across her complete “online life span.” Specifically, we added 1 new feature to the predictive model—time stamp *t*. An instance in the dataset would reflect a user’s historical activities until *t*. As the unit of *t* was the same for all users (a week in our experiment), 1 user could correspond to multiple instances in the dataset. For example, a user who churned in her third week of activities had 3 instances in the dataset—1 instance for her activities and features until the end of her first, second, and third week, respectively. The first 2 instances were labeled as “negative” because the user was still active during these 2 weeks, while the third instance was labeled as a “positive” instance because the user churned in her third week. In other words, the unified model tried to capture the complete life span of a user in the OHC.

To train the unified model, 24,000 users were randomly selected from 47,581 users in the OHC to be included in the training dataset, while others were placed in the hold-out testing dataset. It is worth noting that the unified model with time stamps as a feature greatly increased the amount of training data because a loyal user who had been active for a long time would have many instances in the dataset. However, 24,000 users in the training dataset resulted in 132,341 instances in total. We built the training dataset and trained the model on a high-performance computing cluster. We also made sure that instances for the same user must belong to the same fold in cross-validation.

## Results

### Results of Social Support Detection

**Figure 1:**
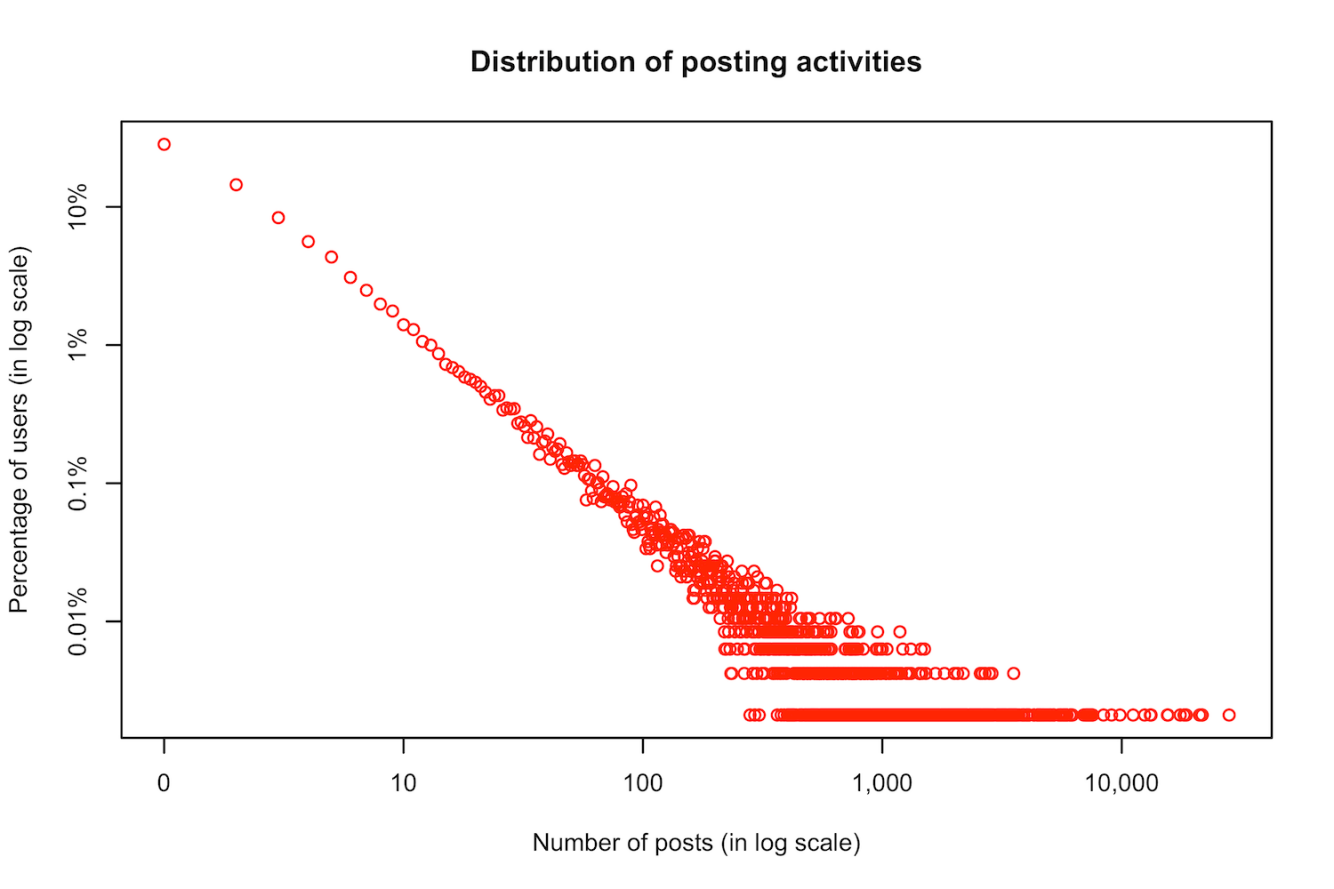
Log-log plot of users’ posting activities in the online health community (OHC).

**Figure 2:**
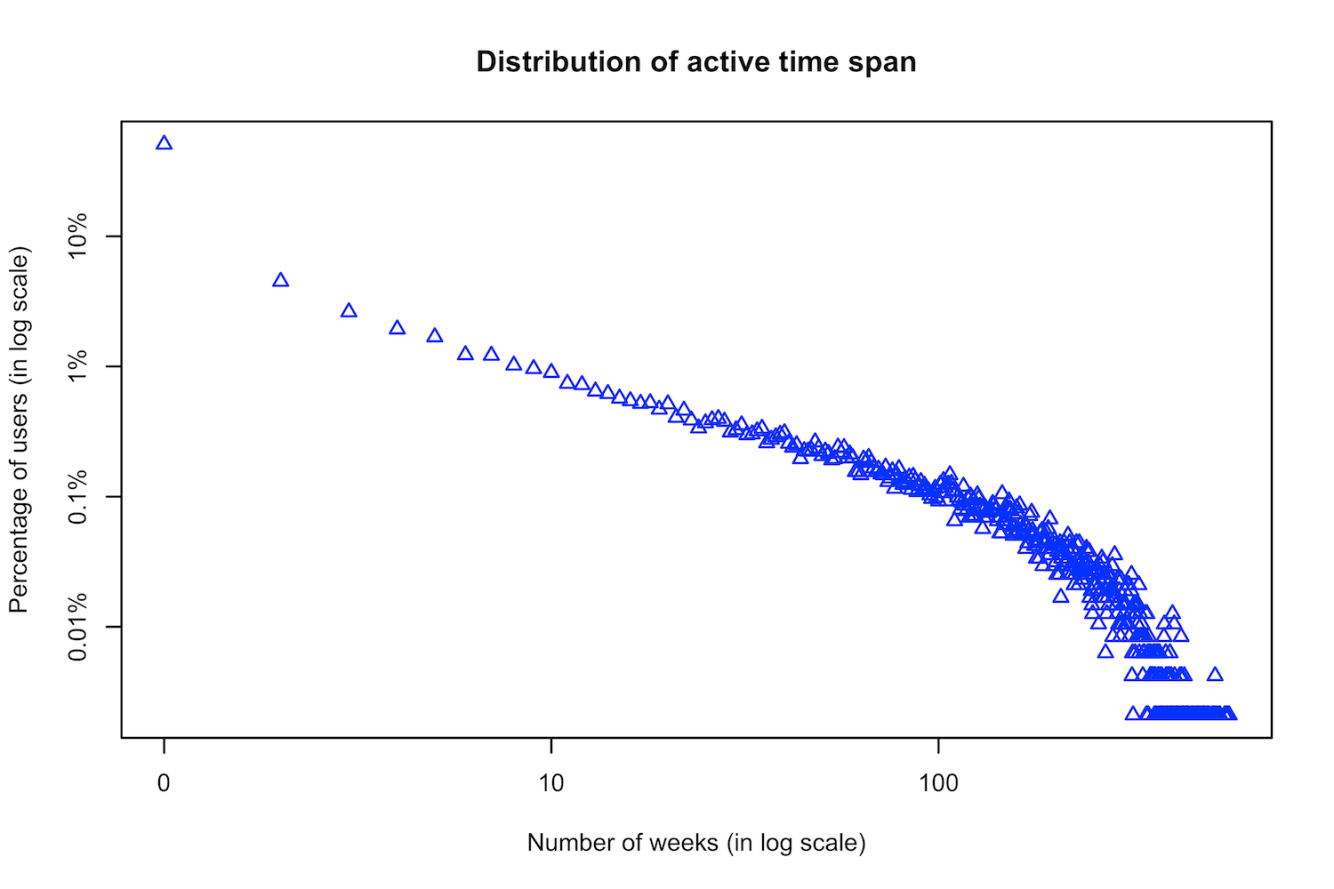
Log-log plot for the time span of users’ online posting activities.

In our dataset from Breastcancer.org, there were more than 2.8 million posts contributed by nearly 50,000 users, including 107,549 initial posts. Figures 1 and 2 show the distribution of the number of published posts and the time span of users’ posting activities in this OHC. The 2 plots indicate that users’ online behaviors featured highly skewed distributions that are similar to power-law distributions. In other words, many users were not very active in posting, while some users were very productive and stayed for a long time.

Because we considered 5 categories of social supports and a post may belong to more than 1 category, we built a classifier for each category. For the classification of each category of social support, we applied various classification algorithms on annotated posts and picked the best performing algorithm using 10-fold cross-validation. Because posts seeking emotional support (SES) accounts for only a small proportion among annotated posts (22 out of 1333), we oversampled posts seeking emotional support when building the SES classifier. Among all the classifiers we tried, AdaBoost, with Naïve Bayesian as the weak learner, was chosen to classify COM, PES, PIS, and SIS, while logistic regression was the best choice for SES (Table 3). Overall, our classifiers achieved decent performance with an accuracy rate of more than 0.8 in all 5 classification tasks.

**Table 3:** Performance of classification algorithms for 5 categories of social support.

Social support	Results	Naïve Bayesian	Logistic regression	Support Vector Machine (polynomial kernel)	Random forest	Decision tree	AdaBoost
COM^a^	Accuracy	.696	.787	.783	.771	.767	.804^f^
	AUC	.839	.817	.768	.848	.75	.852^f^
PES^b^	Accuracy	.713	.830	.840^f^	.830	.81	.817
	AUC	.823	.787	.681	.825^f^	.687	.817
PIS^c^	Accuracy	.753	.813	.823^f^	.767	.779	.801
	AUC	.824	.83	.783	.837	.717	.859^f^
SES^d^	Accuracy	.893	.901	.970^f^	.967	.963	.963
	AUC	.749	.867^f^	.656	.851	.671	.668
SIS^e^	Accuracy	.851	.880	.943^f^	.931	.937	.914
	AUC	.893^f^	.803	.745	.86	.766	.869

^a^COM: companionship.

^b^PES: providing emotional support.

^c^PIS: providing informational support.

^d^SES: seeking emotional support.

^e^SIS: seeking informational support.

^f^denotes the best performer for each row.

**Table 4:** Total numbers of posts in each category of social support.

Social support category	Total number of posts
Companionship (COM)	932,538
Seeking informational support (SIS)	284,027
Seeking emotional support (SES)	227,188
Providing informational support (PIS)	1,034,682
Providing emotional support (PES)	497,096

### Results of Participation Analysis

Table 5 shows the results of the participation analysis based on Cox Proportional Hazard models. Variables with hazard ratios lower than 1 contributed positively to the “survival” (ie, continued participation) of users, whereas those with hazard ratio higher than 1 were considered “hazardous” to keep users in this OHC. Three independent variables (PIS, SES, and COM) had hazard ratios that were lower than 1, meaning that users who provided more informational support, sought more emotional support or posted more companionship had longer time spans of activities in the OHC. More specifically, a hazard ratio of 0.919 for companionship meant that a user’s “survival” rate after 1 month was 8.1% higher (100%-91.9%) if her number of companionship posts was one standard deviation higher than the average. In contrast, those who sought or received more informational support (SIS, RIS_D_, and RIS_I_) often left the OHC earlier. Other variables were not significant in the experiment (eg, PES).

### Results of Churn Prediction

We measured the performance of predictive classifiers using standard metrics for classification, including precision, recall, F1 score, and area under the receiver operating characteristic curve (AUC). After comparing the performance of different classification algorithms (Naïve Bayes, logistic regression, and SVM with polynomial kernel) with 10-fold cross-validation on the training set, logistic regression emerged as the best performer for the unified model. As shown in Table 6, the model offers very good performance in predicting churn during the first week. Although recall of the positive class (ie, leaving the OHC) decreased for prediction in later weeks, the precision was still higher than 0.8, and the overall performance measured by AUC was still more than 0.9.

**Figure 3:**
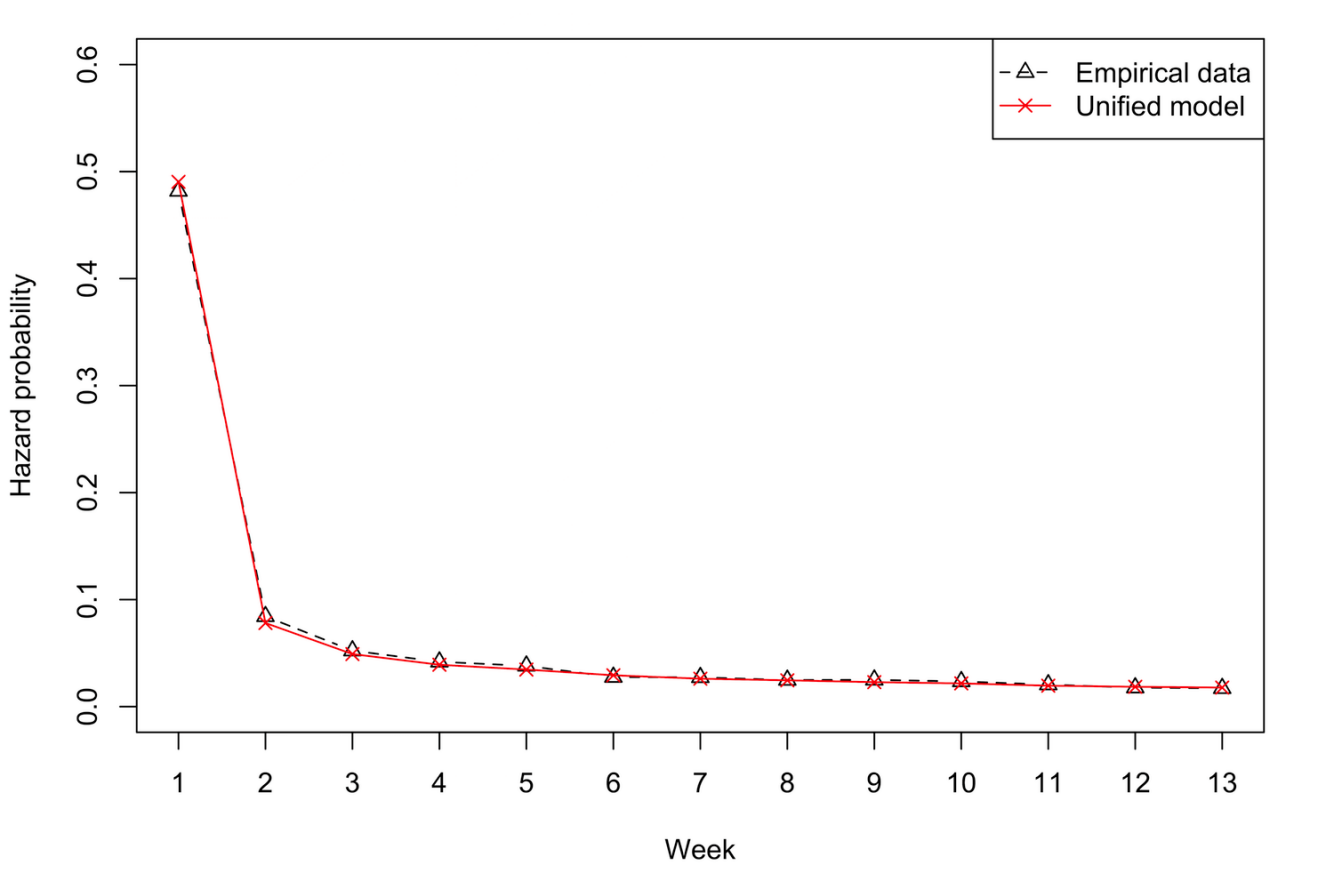
Empirical and predicted hazard curves for user participations.

We also plotted 2 hazard curves to visualize the model’s performance (Figure 3): one based on empirical data and the other based on predictions from the unified model. The horizontal axis represented weeks, and the vertical axis referred to the probability of users’ churn in specific weeks. The 2 curves were very close to each other, indicating good predictive performance from our model at the community level.

**Table 5:** Results from the survival analysis experiment.

Variables	Hazard ratio	*P* Value
InitPost (control)	.995	.75
PES^a^	1.000	.99
PIS^b^	.948***	.001
SES^c^	.972*	.01
SIS^d^	1.050***	.000
COM^e^	.919***	.000
RIS_D_	1.047*	.02
RES_D_	.997	.79
RIS_I_	1.053*	.02
RES_I_	.964	.11
RCOM	.983	.41

^a^PES: providing emotional support.

^b^PIS: providing informational support.

^c^SES: seeking emotional support.

^d^SIS: seeking informational support.

^e^COM: companionship.

**Table 6:** Performance of the unified model on hold-out testing sets in different weeks (precision and recall are for the positive class).

Measures	Churn in the 1st week	Churn in the 3rd week	Churn in the 5th week	Churn in the 13th week
Precision	.950	.872	.880	.838
Recall	.937	.534	.511	.504
F1 score	.943	.662	.647	.629
AUC^a^	.972	.901	.909	.929

^a^AUC: area under the receiver operating characteristic (ROC) curve.

## Discussion

### Principal Findings

The results of survival analysis showed that seeking or providing various types of social support was related to users’ participations in different ways. First, informational support is the most popular social support being sought and provided. This was expected for communities based on common social identities [59] because the large amount of information about a disease and the common identity as patients of the disease are probably why many users come to the OHC in the first place. While providing more informational support is positively correlated with longer participations, seeking and receiving informational support are negatively associated with participations. In other words, those who focus on seeking information may not stay in the long run, even after they receive informational support.

Second, companionship had the lowest hazard ratio. Recall that companionship includes discussions of offline events, sharing daily life stories, birthday wishes, and playing online games. This is a very interesting finding—even though this is an OHC about cancer, discussions of non–cancer-related issues are the key to keeping users engaged in the community. This highlights the importance of building personal bond [59] through off-topic discussions in the form of sharing personal stories about life or having fun together, which can strengthen the connections among users more than informational support. The role of companionship has significant implications for the management of an OHC. Although some OHCs may discourage off-topic discussions in order to achieve a “cleaner” environment with only relevant content, these discussions turn out to be a good way to bond users and keep them engaged, and OHC managers may want to encourage, or even initiate, more of these activities.

Third, although we expected emotional support to be positively related to user participation as suggested by [24], the results are mixed based on whether emotional support was being sought, provided, or received. The hazard ratio of SES was below 1 in the experiment, which contradicts the effect of SIS and suggests that SES can be a sign of longer participations, especially for those who have been with the OHC for a while. However, providing and receiving emotional support are not significant factors. We conjectured that a fair amount of emotional support in the OHC could be generic and a mere formality (eg, “I will pray for you,” “Love you and Hug”). Such emotional support can still be valuable for those who seek support, but activities in providing and receiving such support are not related to users’ continued participations.

Our survival analysis has shown the effects of social support activities on users’ engagement. How much do such social support activities contribute to the performance of the churn predictive model? To illustrate which features are more important for the unified model’s predictive power, we ranked the 145 features in the model using information gain [60]. Among the top 20 features (Table 7), 11 features among the top 20 were made possible only after our classification of different types of social support. Also, 18 of them were features that reflected the temporal dynamics in users’ social support activities, especially the stability during the last week of the training period. Overall, this shows that users’ activities in seeking, providing, and receiving different types of social support, as well as their temporal dynamics in these activities, can greatly enhance churn predictions in OHCs.

In this research, we mined large-scale data to better understand and predict users’ continued participations in OHCs. We first detected the seeking and provision of different types of social support from OHC users’ posts using text mining techniques. Then, survival analysis revealed that companionship is a significant and positive predictor of users’ continued participations. Not limiting the potential of the large-scale data to descriptive analytics, we also developed a churn prediction model with high accuracies. Our work serves as an example that highlights the power of data analytics in exploring complex human behaviors.

From a managerial perspective, the outcome of our study can provide OHC managers with suggestions on how to sustain users’ participations and decision support to retain users through interventions (eg, post recommendations and email reminders). A sustainable and successful OHC will eventually benefit its users. From a methodological perspective, this study was the first to use text mining to differentiate the seeking and providing of various types of social support from large-scale OHC data, and demonstrated how such detection of social support activities could help to understand and predict users’ engagement in OHCs.

This study has practical implications. Traditionally, an OHC will send reminder emails to a user who has been inactive for a while, hoping to raise the user’s interests in coming back. With the help of our churn prediction model, an OHC can find at an early stage whether a user is about to leave. Then, it can intervene proactively and try to retain the user via email reminders. More importantly, instead of including a generic reminder or some random recent posts from the community, such emails can be designed based on the results of our survival analysis. For example, because companionship is a key predictor of users’ continued participations, including some of these companionship posts (eg, birthday wishes, holiday plans, and online scrabble games) in reminder emails may be more effective to keep users engaged than having random posts or just informational posts.

**Table 7:** Top 20 features by information gain for the full unified model.

Rank	Feature
1	Stability of the total number of threads a user initiated during the last week of the training period
2	Stability of the number of threads a user participated during the last week of the training period
3	Stability of the number of SIS^a^ a user posted during the last week of the training period
4	Stability of the number of SES^b^ a user posted during the last week of the training period
5	Stability of the total number of posts from a user during the last week of the training period
6	Stability of the number of PIS^c^ a user posted during the last week of the training period
7	Stability of the number of PES^d^ posts a user received directly during the last week of the training period
8	Stability of the number of PES a user posted during the last week of the training period
9	Stability of the number of COM^e^ a user posted during the last week of the training period
10	Stability of the number of PIS posts a user received directly during the last week of the training period
11	Stability of the number of COM a user was exposed to during the last week of the training period
12	Stability of the number of PES posts a user received indirectly during the last week of the training period
13	Stability of the number of PIS posts a user received indirectly during the last week of the training period
14	Total number of posts from a user during the last week of the training period
15	The number of threads a user participated in during the last week of the training period
16	Stability of the number of threads a user participated in across weeks
17	Stability of the total number of posts from a user across weeks
18	Entropy of the total number of posts from a user during the last week of the training period
19	Stability of the number of PIS posts a user received indirectly across weeks
20	Stability of the total number of threads a user initiated across weeks

^a^SIS: seeking informational support.

^b^SES: seeking emotional support.

^c^PIS: providing informational support.

^d^PES: providing emotional support.

^e^COM: companionship.

### Limitations

This study also had limitations. First, for the 3 independent variables for indirect support received (*RIS*_I_*, RES*_I_*, and RCOM*), we assumed that a user received indirect support when she replied to a thread initiated by another user and read other users’ replies to the thread. This approach of capturing indirect support received could be inaccurate: on one hand, we might underestimate the amount of support because we limited our calculation to threads a user replied to, while a user could get indirect support by reading a thread without posting a reply. On the other hand, our approach might also overestimate such indirect support because when posting to a long thread, a user might not have time to read all the previous replies. This limitation can be addressed by analyzing users’ click streams, but such data were not available for this study and can be difficult to obtain for many studies of OHCs. The lack of clickstream data also prevented us from analyzing lurking behaviors, which might also provide social support to lurkers [35]. Having users’ clickstream data will also help us better define each user’s temporal span of online activities. Second, our survival analysis only reveals the correlation between users’ social support activities and their participations, without showing any causality. Although randomized experiments are better choices to infer causality, there might be ethical concerns to run such experiments in OHCs (eg, keeping certain users away from social support). Alternative approaches are needed to identify causal relationships. Last but not least, our study was based on data from one OHC for breast cancer. In OHCs for other diseases, especially acute diseases (eg, flu), social support activities and users’ engagement patterns may differ. Although the specific results we found for this breast cancer OHC may not be applicable for all OHCs, the framework of methods we used to classify social support and analyze users’ continued participation based on social support activities can be applied to other OHCs.

### Future Work

There are several interesting directions for future research. Detecting users’ health status from their posts will be an interesting endeavor, as it not only can help understand why a user leaves an OHC, but also can potentially improve the recommendation and retrieval of Web-based information. We are also interested in improving the unified predictive model, which is easier for OHCs to use. One possible way is to rebalance instances in the unified model’s dataset because the current dataset features way more negative instances than positive. It would also be interesting to explore whether users’ engagement behaviors change over time, especially when accessing the Web using mobile devices is becoming more popular in recent years. We would also like to collaborate with OHC operators to evaluate the effectiveness of interventions aiming at keeping users engaged.

## Appendix

Multimedia Appendix 1 Post tagging guidelines and outcomes.

Multimedia Appendix 2 Features engineering for social support classification.

Multimedia Appendix 3 Hazard model.

Multimedia Appendix 4 Features engineering for the churn predictive model.

## Abbreviations

AUC: area under the receiver operating characteristic curve
COM: companionship
OHC: online health community
PES: providing emotional support
PIS: providing informational support
SES: seeking emotional support
SIS: seeking informational support
SVM: support vector machine
TV: temporal variations
