# Supplementary material for: Analyzing and Predicting User Participations in Online Health Communities: A Social Support Perspective
Source: J Med Internet Res. 2017 Apr 24;19(4):e130. doi: 10.2196/jmir.6834 (PMC5422656; doi:10.2196/jmir.6834)
Supplement: Multimedia Appendix 1 [file jmir_v19i4e130_app1.pdf]

Your task is to annotate posts from a forum among breast cancer survivors. You are given a set of posts, including initial posts and comments in threaded discussions. For each post, you will need to decide whether the post is related to the following types of social support.

- Seeking informational support
- Providing informational support
- Seeking emotional support
- Providing emotional support
- Companionship
- Could not tell

Types of Social Support

- **Informational support**

  Definition: Informational support is the transmission of information and guidance. The content of the post is related to advice, referrals, teaching and personal experience with treatment or symptoms, which might include medical information for the patients. Such support can help users get better knowledge on how to handle their conditions.

  Seeking informational support: A user posts the message to seek information support, such as asking questions for treatment.

  Examples:

  - I had surgery Aug05 for bc recurrance. B4 surgery I had 33 IMRT rads, prior to that had 4A/C & 4 Taxol. I had bc in 2000 & had 37 rads in same general area. Now, my surgery won't heal. Wound doc says there is adema or something on my sternum (shown on recent MRI). My wound has been draining since it broke open in Sept. Drainage was clear, yellowish & some blood. Now, drainage is milky. some healing is taking place, but Wound Doc says healing may stall cause rads are the gift that keeps on giving. Anyone else been here? Trudan
  - Anyone successful with weaning off Ativan. At first I was taking .5mg at night but when I started Taxol, I'm so anxious I'm now taking 2mg at night. One tablet at about 6:00pm and the other around 9:00pm. I've tried taking just 1 mg, but wake up wide awake with rapid heart rate.
    Anyone with a successful formula for weaning off.

- Where do you buy digestive enzymes and what are they called?
- I am wondering why the doctors are letting so much time go by…

Providing informational support: A user posts the message to provide information support, such as self-description about the experience of illness/treatment.

Examples:

- Wonderful news! Good Morning America featured a segment that a study of 20,000 post-menopausal women discovered that an arthritis drug - Evista (Raxolfine) had proven even more effective than Tamoxifen as a prevention for breast cancer. It has also been shown to have fewer side effects. It needs to be approved by the FDA but they expect it to be approved within the next few months. Catherine
- Hi, I wear a sleeve and glove about 7 hours a day, and I do use a rubber glove to put them on simply because it's easier! I wash the sleeve every few days, the glove less often. That's a matter of personal choice, I guess. Still wrap at night with the stockingnette, Isotoner glove, cotton padding and then an elasticized 'tubular' sleeve. My therapist thought that would be fine for me without going to the trouble of the Reid Sleeve. Never had a pump!
- I took Xanax and Ativan for the anxiety, morphine for the pain, Ambien to sleep at night.
- Dr. Susan Love has a good book about it and probably a website.
- I'm not a Sept surg, bit did have a lumpectomy and sentinel node biopsy on Friday. I'm feeling pretty good. Was able to have a shower today and take all of the big bandages off. Just have the steri strips left. I was out for a couple of short walks yesterday and did an hour on the treadmill this morning (I'm a big walker and was itching to get going). Pain has not been too bad. I'm glad to have surgery over. Now I'm nervous about the final pathology. I get those results the same day you have surgery. Good luck. Hope everything goes well for you.

- **Emotional support**

Definition: Emotional support is transmitted through sharing poignant events. Content contains affection, affirming, validation, sympathy, caring and concern etc., which can help the patients reduce their levels of stress, such as the expression of understanding, the empathy that the patient is not alone, and the post of encouragement that the patient could get better with appropriate care.

Seeking emotional support: The user posts the message to seek emotional support, such as mood description.

Examples:

- I'm just glad I ended up with such a great breast surgeon. The biopsy itself was a bit uncomfortable but the local anesthesia helped a lot. I got a little startled whenever I'd hear that loud "stapler" sound. But she was quick and got good samples. My axillary node looked a bit suspicious so she took a sample from there as well. So now begins the wait! She said hopefully I'll have results by Friday but if not, it will be Monday at the latest. I really don't know if I can go an entire weekend without a diagnosis. I really pray I get the information Friday!
- Strong family history of cancer , just found out I'm getting tested in December for the brac gene , I have to see a counsellor first all sounds so scary to me . I lost my mum to breast cancer it's already bringing back horrible memory's ...
- They say hopefully it will buy us a year but there is no hope of a cure.
- I just told her like it is, no holding back on her. Explained that she can continue to keep her head in the sand and depend on others to do their job, or take control of her own life and by the way didn't she need to do her will? I was not a softy with her, no coddling.
- No kidding about how you can go from the top of the ride to nearly crashing.

Providing emotional support: The user posts the message to provide emotional support, such as using blessing words to pray for others.

Examples:

- hello Julie, I hope you got to sleep and don't get to read this until late morning! Just wanted to let you know that someone has read your post, and to welcome you to the boards, even though I wish we'd met under better circumstances.
- For now, take a deep breath. We've all been through what you're experiencing now, but I promise you, once you have a full dx and a treatment plan in place, things will be much easier to handle. I, too, am 42 yrs old, with 3 kids. It's not easy, but you'll find a way to cope with your 'new normal'. You ARE strong!
- Hang in there,
- Hugs, Nicky

- I just found out the results of my biopsy yesterday and cancer was also found. Like you I am just in shock and scared. My MRI is next week to help determine the treatment. Like you I don't want to talk to my family and husband all the time about this although they are loving and supportive (but don't all know yet!). All I can say to you for now is you are not alone and reading all the posts on here certainly proves that. We will get through this!
- I'm sorry to hear your news.
- Come here anytime to vent, ask questions, cry, and most of all learn from many other women who have "been there, done that".
- ((((((((((((hugs)))))))))) to you.
- Missing Patti. And realizing that this thread needed a gentle bump ~ Thinking of all of us here today who have had a tough day, a sad day, a confused day..those who are making the most of each day. Bless us all.
- I've seen people beat this before.

- **Companionship**

Definition: Companionship helps to strengthen group members' social network and sense of communities. Companionship consists of chatting, humor/teasing, offline events, and groupness (participation in the group, join or respond to the group). Patients share everyday events and ideas such as cooking, gardening, travel, weather, holiday, birthday, anniversary, bingo/scrabble, leisure activities, etc.

Examples:

- Like many people, I have lost some family members to cancer. This year I'm participating in the Enbridge Ride To Conquer Cancer. It is a 2 day - 200Km cycle from Toronto to Niagra Falls! I'm doing this to raise funds, create awareness, and to show my support for those battling this disease. I'm posting this message here as an awareness, just to let you all know that people care, and that we are doing something about it. If you wish to learn more about it, or support these efforts, you may click on link below, and feel free to PM me anytime.
- As the title says, I'm on my way!!! I will use this thread to update all of the goings on over the next six weeks until I return home. I will be posting pics also from time to time so everyone can travel with me. First instalement tomorrow from London as my laptop battery is about to die.......I knew I'd forget something!!!!!! Love n hugs all. Chrissy

- I hope no one is offended by this one. : FW: Sister Mary Katherine, Sister Mary Katherine entered the Monastery of Silence. The Priest said, ‘Sister, this is a silent monastery. You are welcome here as long as you like, but you may not speak until I direct you to do so.’; Sister Mary Katherine lived in the monastery for five years before the Priest said to her, ‘Sister Mary Katherine, you have been here for five years. You may speak two words.’ Sister Mary Katherine said, ‘Hard bed.’ ‘I’m sorry to hear that,’ the Priest said, ‘We will get you a better bed.’ After another five years, Sister Mary Katherine was called by the Priest. ‘You may say another two words, Sister Mary Katherine.’ ‘Cold food,’ said Sister Mary Katherine, and the Priest assured her that the food would be better in the future. On her 15th anniversary at the monastery, the Priest again called Sister Mary Katherine into his office. ‘You may say two words today.’ ‘I quit,’ said Sister Mary Katherine. ‘It’s probably best,’ said the Priest. ‘You’ve done nothing but bitch since you got here.’; Niki
- Calling all Bay Area Babes (I hope no one is offended by the phrase) to see if any local gals might be interested in meeting on a weekend afternoon. You can read some of our postings under "Any Ladies from the SF Bay Area?/ Help Me Get Through Treatment").
- Heading to Chicago and Iowa City
- Iowa City -- July 18 and 19 -- Homewood Suites --- supporting our SISTER Mcook http://coasttocoastforcancer.org/michelleb/
- Let us know how it goes.
- You know, I watched an old movie to get into the spirit of the snowfall last night.
- Happy birthday Marty and if you go streakin don’t forget to iron your Birthday Suite!!

You will use binary options (Y or N). For example, a “Y” on the “seeking informational support” column for a post means you agree that the post tries to seek informational support. Similarly, an “N” on “providing emotional support” means you believe this post is not providing emotional support.

Note that some posts could be related to more than one type of support. For example, a post could be tagged "Y" for both seeking informational support and seeking emotional support, which means the user posts the message to seek two kinds of support.

Examples:

Seeking both information support and emotional support:

- I felt a lump on my breast which was about 2.5cm. I was not sure how long it was there. Had mammogram diagnostic and ultrasound, then they referred me to see a breast surgeon. I had a lumpectomy on Oct. 8 and on Oct 10 they called me and told me they found cancer in the lump. My surgeon was shocked and I felt scared and sad. I have a 5 year old boy who I planned to see him grow up. I am 34, and why this is happening to me! So far they did not have all the information to tell me how serious it is. They will probably contact me again next week. I talked to a nurse navigator, she told me I might get more surgery first or chemo first. I feel so helpless out here. I have a loving family, but i do not want to talk about this with them all the time. Please help!

Providing both information support and emotional support:

- So sorry you are going through this. I remember all too well how scary those early days were. You need to remember to breathe (hard to do i know) You will know more once you talk to the doctor to see what the indicators are for the cancer they found. Once they do ask for a copy of your pathology report and go to the main BCO page (and here as well) and you will see what you are dealing with. Remember to come here to rant, rave and talk to others that are in the same walk as you are. Prayers to you as you begin your journey.

Please

- Use the definition and examples above to determine the tags.
- Use all your knowledge of online social communities to guide your decisions.

You might see a string with symbols, for example, "((((Sherry)))))", it could be an emoticon.

If you have any question or concern, please do not hesitate to let us know.

Table A1. The number of posts in each category of social support in the annotated dataset.

Social Support Category	Number
Companionship (COM)	435
Seeking Informational Support (SIS)	96
Seeking Emotional Support (SES)	22
Providing Informational Support (PIS)	411
Providing Emotional Support (PES)	249
