# Supplementary material for: Analyzing and Predicting User Participations in Online Health Communities: A Social Support Perspective
Source: J Med Internet Res. 2017 Apr 24;19(4):e130. doi: 10.2196/jmir.6834 (PMC5422656; doi:10.2196/jmir.6834)
Supplement: Multimedia Appendix 2 [file jmir_v19i4e130_app2.pdf]

Table A2 summarizes these features. Many of the features were picked specifically for classification in this context. For example, we included “whether a post is an initial post” as a feature because many users seek support by starting a thread. Inside a post, the existences of URLs and emoticons are often related to informational and emotional supports respectively. Similar to the approach used by Wang et.al [1], we also checked the usage of phrases in the format of <you/he/she + MODAL verb > to express possibilities, such as “you should”, “she could”. We considered “he” and “she” in addition to “you”, because some posts were created by family members of cancer survivors. To identify the difference between “seeking” and “providing” support, we included words related to seeking behaviour, such as “question”, “wonder” and “anybody”. We also hoped that words related to daily life topics and geographical locations can effectively detect companionship.

Meanwhile, we used OpinionFinder [2] to find the overall sentiment, as well as subjectivity and objectivity of each post. Besides these hand-picked or dictionary-based lexicons, we also wanted to capture whether the usage of other words and phrases can contribute to the classification. Using unigrams and bigrams is too fine-grained and leads to a feature set with very high dimension. Thus we also applied the topic-modelling technique Latent Dirichlet Allocation (LDA, with k=20) [3] to the content of all posts and generated 20 topics. For each post, LDA gave a topic probability distribution, indicating the probability of this post corresponding to each topic. Such a distribution for each post was then included in the feature set.

Table A2 Summary of features for the classifier.

Group	Features
Basic	Whether the post is an initial post in a thread

Features	Whether the post is a self reply
	Length of the post
Lexical Features	Whether the post contains URLs (Y or N)
	Whether the post contains emoticon(s)
	Number of numeric numbers
	Number of Pronouns (e.g., they, we, I)
	Whether the post contains the negation word(s) (e.g., not, never, no)
	Whether the post contains name(s) of city, state, country (U.S.A, Canada, etc.)
	Whether the post contains phrases related to possibility (you must, you might, she had better, etc.)
	Whether the post contains names of drugs related to breast cancer (From http://www.cancer.gov/cancertopics/druginfo/breastcancer)
	Whether the post contains breast cancer terminology (From http://www.breastcancer.org/dictionary)
	Whether the post contains verb related to advice (Need, require, recommend, etc.)
	Whether the post contains emotional words (Love, sorry, hope, worry, etc.)
	Whether the post contains words related to seeking behaviours (Anybody, question, wonder, etc.)
	Whether the post contains words related to daily life topics (Vacation, joke, run, walk, etc.)
Sentiment Features	Frequency of words with positive and negative sentiment
	Objectivity and subjectivity scores
Topic Features	Topic distributions derived from LDA

References

1. Wang Y-C, Kraut R, Levine JM. To stay or leave?: the relationship of emotional and informational support to commitment in online health support groups. Proc ACM 2012 Conf Comput Support Coop Work [Internet] New York, NY, USA: ACM; 2012 [cited 2013 Jul 12]. p. 833–842. Available from: http://doi.acm.org/10.1145/2145204.2145329

2. Wilson T, Wiebe J, Hoffmann P. Recognizing Contextual Polarity in Phrase-level Sentiment Analysis. Proc Conf Hum Lang Technol Empir Methods Nat Lang Process [Internet] Stroudsburg, PA, USA: Association for Computational Linguistics; 2005 [cited 2016 Dec 1]. p. 347–354. Available from: http://dx.doi.org/10.3115/1220575.1220619

3. Blei DM, Ng AY, Jordan MI. Latent dirichlet allocation. J Mach Learn Res 2003;3(Jan):993–1022.
