# Supplementary material for: Analyzing and Predicting User Participations in Online Health Communities: A Social Support Perspective
Source: J Med Internet Res. 2017 Apr 24;19(4):e130. doi: 10.2196/jmir.6834 (PMC5422656; doi:10.2196/jmir.6834)
Supplement: Multimedia Appendix 3 [file jmir_v19i4e130_app3.pdf]

The hazard $h_i(t)$ represents the events occur to individual i at time t (defined in Equation 1),

$$h_i(t) = h_0(t) * \exp\{\beta_1 x_{i1} + \beta_2 x_{i2} + \cdots + \beta_k x_{ik}\} \quad (1)$$

where the baseline hazard function $h_0(t)$ can be any function of time t as long as $h_0(t) > 0$. x_i and β_i represent independent variables and corresponding coefficients. Equation 1 can also be formulated as Equation 2, where the ratio of two individuals' hazard functions does not depend on time t.

$$\frac{h_i(t)}{h_j(t)} = \exp\left\{\beta_1(x_{i1} - x_{j1}) + 1 + \beta_k(x_{ik} - x_{jk})\right\} \quad (2)$$

By using Maximum Likelihood Estimation, β can be estimated with regards to the hazard. $\beta_k = 0$ would indicate that independent variable x_k has no association with survival time; $\beta_k > 0$ means that independent variable x_k induces a higher hazard of event occurring, and vice versa. Correspondingly, $\exp\{\beta_k\}$ is the hazard ratio of independent variable x_k.
