# Supplementary material for: Analyzing and Predicting User Participations in Online Health Communities: A Social Support Perspective
Source: J Med Internet Res. 2017 Apr 24;19(4):e130. doi: 10.2196/jmir.6834 (PMC5422656; doi:10.2196/jmir.6834)
Supplement: Multimedia Appendix 4 [file jmir_v19i4e130_app4.pdf]

We included the following features to capture temporal dynamics of users' online activities.

(1) The overall slope of a feature—a positive slope suggests a user's weekly activities was on the rise during the training period, and vice versa;

(2) The Shannon entropy [1] of users' weekly activities, with lower entropy indicating more stable activities for the corresponding feature during the training period. For instance, if a users' total number of posts across 4 weeks are 1, 2, 1, and 3 respectively, the probability of publishing 1 post in a week is ½. The probabilities of publishing 2 posts in a week is ¼, and so is the probability of publishing 3 posts in a week. Based on Equation 3, the entropy of total number of posts published would be $-\left(\frac{1}{2}*\log_2\frac{1}{2}+\frac{1}{4}*\log_2\frac{1}{4}+\frac{1}{4}*\log_2\frac{1}{4}\right)=1.5$. However, this metric only considers the appearance of different numbers, instead of numeric values of these numbers. For instance, another user with 1, 5, 1, and 6 posts in 4 weeks will have the same entropy as the previous user with 1, 2, 1, and 3 posts.

$$Entropy=-\sum p*\log p \tag{3}$$

(3) The new metric of stability is used to address the problem of Shannon entropy. Its calculation is similar to Shannon entropy, as defined in Equation 4, but p_i' represents the proportion of activities from week *i* compared to the total activities from all weeks. The higher the stability metric is, the more stable a user's activities over time.

$$Stability=-\sum p_i'*\log p_i' \tag{4}$$

To handle cases when all the values are 0 during a time period, we also adopted Laplace Smoothing (a.k.a., Add-one Smoothing). The same example for Shannon Entropy is used to illustrate how stability is calculated. Note that the total activities are *1+2+1+3=7*.

$p'_1=(1+1)/(7+4)$, $p'_2=(2+1)/(7+4)$, $p'_3=(1+1)/(7+4)$, $p'_4=(3+1)/(7+4)$, and the stability for this user across 4 weeks would be

$$-\left(\frac{2}{11}*\log_2\frac{2}{11}+\frac{3}{11}*\log_2\frac{3}{11}+\frac{2}{11}*\log_2\frac{2}{11}+\frac{4}{11}*\log_2\frac{4}{11}\right)=1.936$$;

(4) The temporal variation (TV) of features, which extends entropy and stability by considering the fluctuation in a temporal sequence of data [2]. For instance, if two users' values of a feature across 4 weeks are 1,3,1,3 and 1,1,3,3 respectively, they will share the same Shannon entropy and stability while the second user has less fluctuation on this feature. User *i*'s TV on feature *f*, is defined in Equation 5, where $f_{i,t}$ measures user *i*'s activity (e.g., total number of posts) during time interval t ; S_i and E_i are the starting and ending time of the training period. Basically, TV measures the average variation between successive time intervals (e.g., weeks) during a given time period (e.g., a month), normalized by the average value across the given time period. The higher the value of TV, the more fluctuated a temporal sequence is.

$$TV_{f,i}=\frac{\frac{1}{E_i-S_i}\sum_{t=S_i}^{E_i-1}\left|f_{i,t}-f_{i,t+1}\right|}{\frac{1}{E_i-S_i+1}\sum_{t=S_i}^{E_i}f_{i,t}} \quad (5)$$

References

1. Shannon CE. A Mathematical Theory of Communication. Bell Syst Tech J 1948 Jul 1;27(3):379–423.
2. Zhao K, Kumar A. Who blogs what: understanding the publishing behavior of bloggers. World Wide Web 2013 Nov 1;16(5–6):621–644.
